# Vision Diagnostics and Treatment System for Children with Disabilities

**DOI:** 10.1155/2018/9481328

**Published:** 2018-01-10

**Authors:** Pawel Kasprowski, Katarzyna Harezlak

**Affiliations:** Institute of Informatics, The Silesian University of Technology, ul. Akademicka 16, 44-100 Gliwice, Poland

## Abstract

Vision plays a crucial role in children's mental development. Therefore, early diagnosis of any vision disparities and implementation of a correct therapy is very important. However, carrying out such a procedure in case of young children and especially children with brain dysfunctions poses some limitations due to cooperation problems. The vision diagnostics and treatment (VisDaT) system presented in this paper is meant to help therapists in proper diagnosis and treatment involving such children. It utilizes a computer connected to two monitors and equipped with a specialized software. The main system components are as follows: an eye tracker recording child's eye movements and a digital camera monitoring online child's reactions. The system is equipped with a specialized software, which creates the opportunity to stimulate children's vision with a dedicated stimulus and post hoc analyses of recorded sessions, which enable making decision as to the future treatment.

## 1. Introduction

From the very moment of birth, every human is faced with the challenge of knowing the world and has to activate all the senses for this purpose. There is a huge progress in skills during the first year of life, and the proper vision development has considerable impact on this process. Recognizing shapes, colors, or other people's emotions is facilitated significantly when the oculomotor system cooperates correctly with the brain. However, not all people are fortunate and their eyesight is impaired from the beginning of their lives or becomes weakened or damaged later. Lack of ability to see the surrounding world properly may result in some serious negative consequences, especially for children, because it may slow down cognitive processes but also influence psychosocial relationships. The problem becomes more severe when it concerns infants or older children whose vision impairments are accompanied by other disabling conditions such as cerebral injuries [1]. The difficulty stems from the fact that a child is unable to communicate what and how he/she sees, which makes reaching a diagnosis a demanding task. It is crucial to evaluate the extent to which children with impairments are able to use their vision, because even weak visual abilities may serve as the basis for vision enhancement [2], which subsequently may entail overall intellectual development [3]. Children with complex disabilities require systematic brain stimulation. If many visual, auditory, and tactile stimuli engage children's attention, then the brain receiving pieces of information from various senses has to organize them by recognizing, analyzing, and integrating them. Therefore, a therapy, appropriate for a particular impairment, should be prepared based on functional vision assessment examination [4]:

- Fixation correctness—ability to keep eyesight on a visual stimulus,
- Eyeballs motility—ability to trace a moving stimulus with eyes,
- Functional visual acuity—a distance from which a child recognizes a character of a given size,
- Contrast sensitivity—the impact of a presented level of a contrast on a child's visual ability,
- Field of vision—an area, within which a child is able to see the presented object.

During series of tests determining conditions, in which a child is able to perceive objects, a feedback from participants is expected. As has been mentioned, it may pose certain difficulties, thus supporting techniques are searched for. The solution that proves useful in dealing with the aforementioned problem is eye tracking technology, which provides methods for registering and analyzing eye movements and thus for evaluating the vision quality.

This was the motivating factor behind the decision to undertake studies on developing an environment for the vision diagnosis and therapy for children with various visual impairments. In this paper, we present such a solution with the discussion of its possible applications. The solution offers using specialized device for collecting eye movements and the multicomponent system facilitating gathering data and its further processing and analysis.

The paper starts with the introduction to eye tracking methods and eye movement data processing presented in Section 2—Eye Tracking Basics. The description of the proposed system including the main modules and algorithms is described in Section 3—VisDaT System. Subsequently, some examples of VisDaT system usages, together with obtained preliminary results, are presented in Section 4—Results. Finally, the last section summarizes the presented studies.

## 2. Eye Tracking Basics

**Figure 1:**
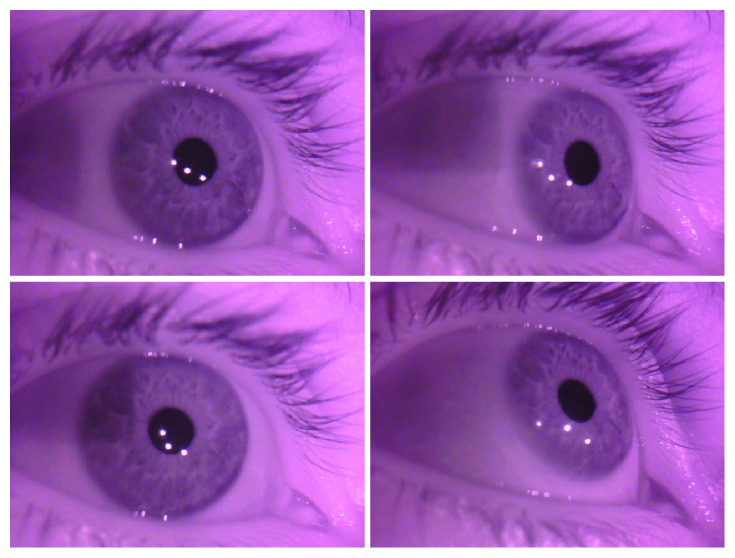
Eye images used by the eye tracker camera. Relation between the three illuminator's reflections and eye center may be used to calculate a gaze point.

When considering an application of eye tracking methods, the first decision must concern a device utilized to record eye movements. Among currently used technologies—electrooculography [5] and video-oculography systems [6, 7]—the latter one, due to its low invasiveness, is the obvious choice for children-oriented experiments. VOG eye trackers do not require direct contact with eyes, because they record eye movement by means of digital video cameras, capturing a sequence of eye images. An eye image may be obtained by the application of an infrared illuminator. Most implementations use near IR light sources with a wavelength of approximately 880 nm, almost invisible for the human eye, but still possible to be detected by most commercial cameras [8]. On the basis of eye images, specialized algorithms are used to evaluate the center of the pupil and the differences in its positions to determine eye movements. Such calculations utilize reflections caused by light falling into the eye (Figure 1).

**Figure 2:**
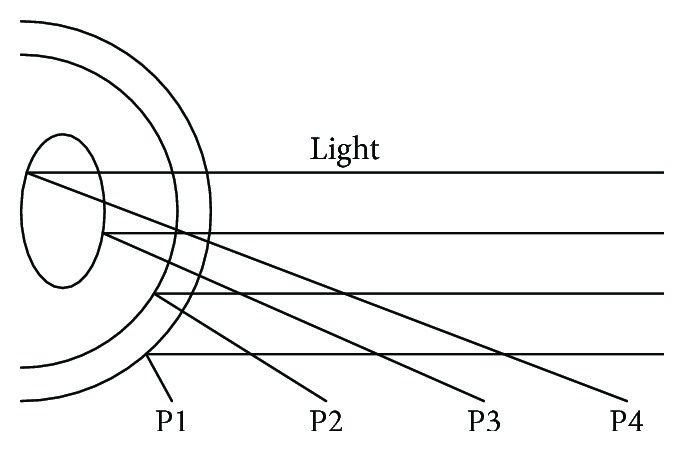
Illustration of four Purkinje reflections. Source: own elaboration.

These reflections are almost stable regardless of an eye ball rotation, which provides reference points for pupil center positions. There are four such reflections named Purkinje images (Figure 2):

- The first Purkinje image (P1) is the reflection from the outer surface of the cornea (CR also called glint).
- The second Purkinje image (P2) is the reflection from the inner surface of the cornea.
- The third Purkinje image (P3) is the reflection from the outer (anterior) surface of the lens.
- The fourth Purkinje image (P4) is the reflection from the inner (posterior) surface of the lens.

Video-based eye trackers usually use only the first one, because this is the brightest and easiest reflection to detect and track. The difference vector between the pupil center and the corneal reflection indicates the direction and scope of eye movement.

The limitation of such a solution is that the eyes should remain visible in the recording, because subsequent eye positions are evaluated based on information obtained from consecutive images. Construction-wise, VOG eye trackers can be divided into three categories:

- *Tower eye trackers*—which use a tower-based ergonomic chin rest to ensure the stable position of the head and record an eye position using a high-end camera located directly above patient's eyes. They ensure the best accuracy and precision among all types of eye trackers.
- *Remote eye trackers*—contactless, placed at some distance in the front of an examined person. Because of this distance, such eye trackers are less accurate but also less cumbersome for users as there is no chin rest and limited head movements are allowed.
- *Glasses*—mobile, wearable eye trackers designed to capture visual behavior in any environment. Such eye trackers are typically equipped with two cameras—eye and scene cameras—and allow users to make free movements. The data obtained from both cameras must be synchronized to calculate the gaze position.

Since we need to ensure that the eyes are seen by an eye tracker camera, a tower eye tracker seems to be the best solution. However, it is easy to imagine how difficult it may be for children, even in the case of healthy ones, to sit in a still position with an immobilized head. It is even more complicated when the problem regards children with various impairments. On the other hand, head-mounted eye trackers allow for free head movement during recordings; however, they may be unadjusted to children's heads, which makes it uncomfortable and disturbing for them. There are trials of overcoming this problem with the device suitable for children [9], but it must be emphasized that an experiment conducted with such a device is rather child driven, because the scene viewed by a child is under its control and it is difficult to automatically correlate a scene camera image with the intended stimulus. Thus, remote eye trackers are considered a solution to be used [10, 11], despite the fact that turning a head away from an eye tracker causes loss of an eye movement signal. The aforementioned problems are strengthened when children with impairments such as cerebral palsy are taken into consideration, because of the lack of ability to control head movements. Thus, an examining setup may be complemented with an additional equipment such as a stroller, car seat, or wheelchair appropriately restricting the infants' movements [12].

### 2.1. Registered Data Processing

Ensuring the possibility of registering eye position is only the first step towards obtaining information about children's vision quality. There are some additional steps, which have to be undertaken to change registered data into valuable information. The aim of the first step is to adjust an eye tracking environment to a particular child, which may be obtained during the calibration process.

The commonly applied calibration procedure consists of two stages. During the first one, the examined person's eye movements are registered when looking at stimuli in known locations of a scene—usually there are points evenly distributed over a screen. In the case of an experiment engaging adults, there are 9 points presented. Subsequently, for correlating an eye tracker output with appropriate points, a function mapping registered eye positions to points of viewed scene is defined. It is further used to provide coordinates of user's gazes (1). Because of idiosyncratic features of the oculomotor system, this process is conducted independently for each examined person [13].

(1) $$\begin{array}{c} x_{s}=f\left(x_{e},y_{e}\right),\\ y_{s}=f\left(x_{e},y_{e}\right), \end{array}$$

where *x*_e_ and *y*_e_ represent the data obtained from an eye tracker and *x*_s_ and *y*_s_ are the estimated gaze coordinates on a screen.

The function *f* may be defined in various ways [14]; however, the solution commonly used is the second degree polynomial regression.

(2) $$\begin{array}{c} x_{s}=A_{x}x_{e}^{2}+B_{x}y_{e}^{2}+C_{x}x_{e}y_{e}+D_{x}x_{e}+E_{x}y_{e}+F_{x},\\ y_{s}=A_{y}x_{e}^{2}+B_{y}y_{e}^{2}+C_{y}x_{e}y_{e}+D_{y}x_{e}+E_{y}y_{e}+F_{y}. \end{array}$$

Not every term of the polynomial must be utilized in the mapping function. It may be adjusted to a particular environment during its configuration, based on the accuracy of provided gaze points. The simplest way for its assessment is the usage of the root-mean-squared error (RMSE), measuring the difference between the stimulus location and the mapped gaze point:

(3) $$\begin{array}{c} RMSE=\sqrt{\frac{1}{n}\sum_{i}{\left(G_{i}\left(x,y\right)-\hat{G_{i}}\left(x,y\right)\right)}^{2}}, \end{array}$$

where *G_i_* is an observed value and $\hat{G_{i}}$ is a value calculated by the mapping function. Nevertheless, it is also possible to apply other methods [15].

Applying the above-presented procedure may be difficult in the case of young children [16], as they lose their attention very quickly and the number of calibration points should be reduced at least to 5 [11]. Sometimes it is even impossible to use so few points, because of the problem with communication or with understanding the rules. It forces us to further decrease the number of points to 2 [9, 12] or the usage of other objects interesting for children. This solution was utilized in the work described in [17], where a small, visually attractive sounding toy was displayed at one of the five predefined spatial positions. Similarly, in [18], infants were shown attention getters placed at the top left and bottom right corners of an imaginary rectangle corresponding to the corners of the stimulus viewed during test. The calibration routine was repeated, if infant's gazes at test stimuli were outside of the assumed spatial accuracy.

However, when children are affected by serious cerebral impairments [4], additional simplification may be required; thus, extended methods are searched for. The purpose of this paper is to present the solution ready to be applied for examining vision quality of children with whom verbal communication is very difficult.

## 3. The VisDaT System Description

An environment meant for a vision diagnosis and therapy, to prove useful, should be equipped with many visual, auditory, and tactile stimuli, which force the brain to organize them by recognizing, analyzing, and integrating them. Additionally, it should be flexible in adjusting to children's needs, which are different dependent on their age and impairment. For this purpose, the VisDaT system was developed in the cooperation with a group of therapists from the BRUNO association involved in daily care of impaired children. They provided many useful guidelines for tool development, especially in terms of children's physical condition and the stimuli elaborating. Based on the collected knowledge, some assumptions have been made with regard to hardware and software configuration. These assumptions and the system implemented under their conditions were described in subsequent sections.

### 3.1. Assumptions

The first and the most important circumstance is the inability to explain and to encourage the group of the previously described children to follow the rules of an intended eye tracking procedure. To facilitate eye movement registration in such difficult cases, it was necessary to prepare the workplace that would be usable even when it was not possible to calibrate users properly. This imposes the need to work on new methods, which could overcome calibration problems by implicit procedure, without any user's cooperation. Even, when the system is not calibrated at all, it should give some feedback—at least information if users somehow respond to presented stimuli.

On the other hand, the system should be attractive for children and engage them as much as possible, taking into account their visual impairments. Therefore, a stimuli preparation should be preceded by an initial recognition of objects which are interesting for a child. It would be convenient to define specific stimuli and feedbacks for each child, that is, specific colors, shapes, and sounds that a particular child normally finds attractive. Additionally, to provide a reward system, visual and acoustic system's responses should be introduced to trigger a proper child's reaction. These responses may be activated automatically or manually by an operator conducting an eye tracking session. It is assumed that this will be a therapist who works with the child on a daily basis.

### 3.2. System Realization

**Figure 3:**
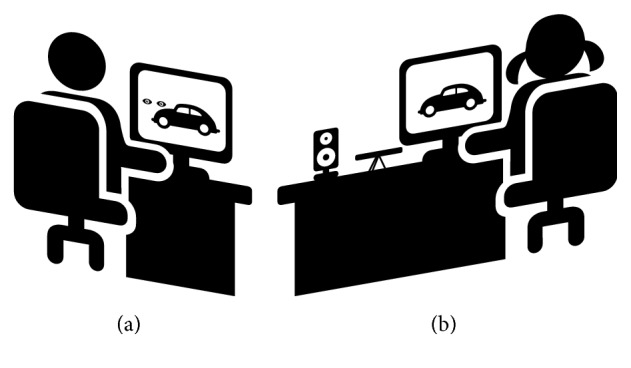
The general view of the VisDat system. It consists of the stimulation screen (b) visible to a subject and the control screen (a) visible to an operator.

The VisDaT system is the extended version of the prototype presented in [19]. It consists of the computer with dual graphics card, two displays, an Eye Tribe eye tracker, Genius Eye camera, and speakers. The examined person (child) sits in front of one of the displays (stimulation screen) and watches stimuli. The Eye Tribe is situated below the display using a special mount and records the child's eye movements during the whole session. The Genius camera is situated above the display and records the child's movements. This information may be helpful in post hoc analysis of the session. Speakers are used to emit sound, which is treated as an award for a child for active participation. The other display (control screen) is located nearby, but in such a way that its content is not visible to the examined person. The operator of the system observes the content of the control screen and uses mouse to change the content of the stimulation screen and to influence the work of the eye tracker. Both outputs—from the eye tracker and the camera—are presented online to the operator. The simplified setup of the system is presented in Figure 3.

The system is controlled by the specialized software. It was written in Java language; thus, it is independent of the operating system. However, so far, it has been tested only in Microsoft Windows 10 environment. The workplace presented in the paper used the Dell Precision T1600 computer with Intel Xeon 3.1 GHz CPU and 4 GB RAM, but any computer with at least one USB3 socket should be suitable.

### 3.3. Solving the Lack of Calibration Problem

As mentioned in Section 2, a typical calibration scenario, when a participant is instructed to follow with his eyes the point displayed on a screen, is not feasible for children with brain disorders as they are not likely to follow any instructions. Therefore, instead of the traditional calibration-presentation scenario, two other techniques were proposed for the presented system: the differential analysis (DA) and implicit calibration (IC).

#### 3.3.1. Differential Analysis

Any usage of an Eye Tribe eye tracker has to be preceded by the calibration to make the registration possible. Thus, at first, it must be calibrated by the operator. Then, the eye tracker's output is calculated based on the operator's calibration and this output is used for subsequent experiments. Such the method does not give accurate absolute gaze coordinates, but it may be used to check, if the eyes move at all.

Our previous experiments have showed that when the eye tracker calibrated for one person is utilized for registering eye movements of other people, in most cases, the direction of a calculated gaze movement is similar, yet less accurate than for the calibrated user [20].

**Figure 4:**
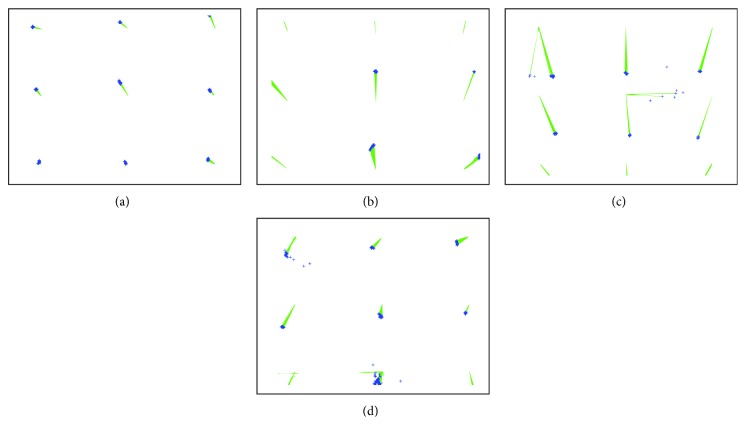
An example of four recordings, when subjects were looking at nine subsequent locations. (a) shows the results for a subject who participated in the calibration, and other three charts (b, c, d) show results for three other subjects using the same calibration model as for the first one.

Examples of such recordings are visualized in Figure 4.  Figure 4(a) shows recordings for the calibrated person, while the following three for other users, evaluated with the usage of the same calibration function. Red crosses denote stimulus locations, the blue ones are the eye tracker's output, and green lines connect calculated gaze coordinates with the actual stimulus positions. It may be noticed that in all cases, eye positions are shifted in one direction, with respect to stimulus. It means that when the same calibration model is applied for different subjects' data should show correct eye movement directions, but not the correct eye positions. Thus, when there is no need to provide a gaze point with the high accuracy, the proposed solution will fulfill its task; namely, it will point out the direction of an eye movement.

**Figure 5:**
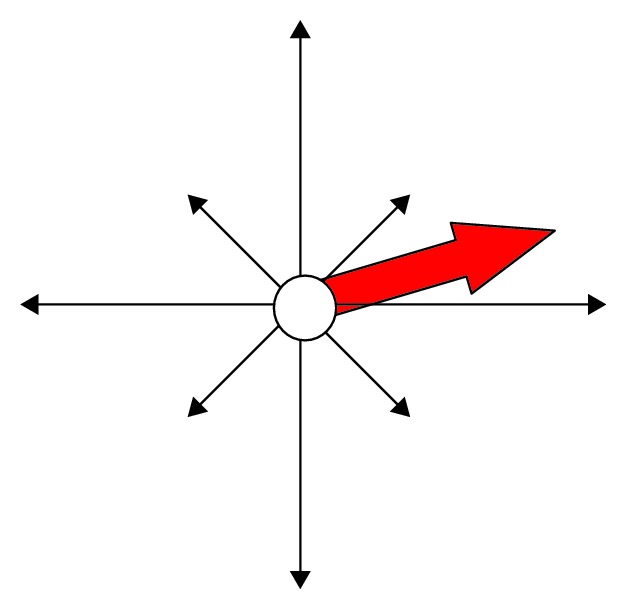
The gaze-rose component. The red arrow shows the current direction of the eye movement.

So, even when the eye tracker is not calibrated for the person who is being observed, it is very likely that real direction of movements will be the same as the one showed by the eye tracker. Therefore, a special component called gaze-rose has been introduced and is visible on operator's screen. The gaze-rose shows the current direction of eye movement (Figure 5).

This direction is presented in the form of a vector (*dx*,*dy*) calculated as a resultant of vectors of movements for the previous five recordings (in a time window of approximately 80 ms of the recording). For a given moment, the *k* coordinates of the vector are calculated using the equations:

(4) $$\begin{array}{c} {dx}_{k}=\overset{k}{\underset{i=k-5}{\sum }}\left(x_{i}-x_{i-1}\right),\\ {dy}_{k}=\overset{k}{\underset{i=k-5}{\sum }}\left(y_{i}-y_{i-1}\right), \end{array}$$

where *x_i_* and *y_i_* are gaze coordinates read from the eye tracker.

The gaze-rose may be used to detect, if a child reacts to a new stimulus. For instance, when the stimulus appears on the right side of the screen and the operator notices sudden eye movement to the right (right red arrow on the gaze-rose), she/he may assume that this movement is a reaction to the stimulus and—what is the most important—that the child sees this stimulus.

#### 3.3.2. Implicit Calibration

As the explicit calibration of the system with children's cooperation is impossible, it must be done implicitly during the normal activity. It requires obtaining some pairs: an eye tracker output—actual gaze coordinates. Given such pairs, it is possible to build a regression function that maps eye tracker output to gaze coordinates. Of course, the function works better when there are more points and the data is of better quality. However, in the case of children with brain disorders, we have to agree on objective restrictions. Therefore, the calibration is done ‘on the fly—when any stimulus appears on a screen and the operator notices any child's reaction (indicated by the gaze-rose)—which may mean that the child is looking towards the stimulus. One click at the operator's screen triggers the process of collecting data for the calibration. The implicit calibration algorithm works as follows:

1. Stimulus appears on the screen.
2. A child moves its eyes towards the stimulus.
3. The operator sees the movement and, when it is finished, clicks on the stimulus to inform the system that the child is now looking at that point.
4. The system starts registration of eye tracker's output and triggers a potential feedback (sound or movie).
5. When eye tracker's output changes (indicating a subsequent eye movement), the calibration module stops the registration and adds the clicked point's coordinates together with the registered eye tracker's output to the existing calibration dataset.
6. The calibration function is recalculated using all data from the calibration dataset.

Of course, more points result in better calibration model, but even one point calibration may be valuable. For instance in [20], it has been shown that even after one point calibration, it was possible to evaluate at which of nine parts of a screen the user was looking.

The number of collected points is not the only factor that influences accuracy. It is also important, if the points are more or less evenly distributed across a screen and if the calibrated area corresponds to the further scene of an experiment. Thus, for instance, if an experiment task is to explore the efficiency of a child vision in the horizontal direction, a stimulus should consist of objects appearing along this axis. Or, if an experiment is based on the central area of a screen, stimuli should be concentrated around this central point within a particular radius.

Another very important factor is the quality of recordings. If the operator clicks the point and the child immediately turns its head or closes eyes, the quality of eye tracker's output could be deteriorated and adding such a point to the model will probably worsen the overall accuracy of the model. Examples of points with high and low deviations may be observed in Figure 4. Therefore, before building the model, the application automatically checks the deviation of eye tracker outputs for each point and then removes points for which the deviation is significantly higher than for others.

The deviation for *N* readings recorded for one gaze point is calculated as follows:

(5) $$\begin{array}{c} \sigma_{x}=\sqrt{\frac{1}{N}\overset{N}{\underset{i=1}{\sum }}{\left(x_{i}-\mu_{x}\right)}^{2}},\\ \sigma_{y}=\sqrt{\frac{1}{N}\overset{N}{\underset{i=1}{\sum }}{\left(y_{i}-\mu_{y}\right)}^{2}}, \end{array}$$

where *x_i_* and *y_i_* are the eye tracker outputs and *μ_x_* and *μ_y_* are the average values in both directions. When there are more than three points registered, the point with the highest deviation is automatically removed from calculations.

The next step is the creation of pairs (*x*_s_,*y*_s_) and (*μ_x_,μ_y_*) where *x*_s_ and *y*_s_ are the screen coordinates and *μ_x_* and *μ_y_* are the average eye tracker's output registered for this point. These pairs are then used to calculate coefficients of the polynomial presented in (2) by means of the classic Levenberg-Marquardt algorithm [21]. Then, the results are immediately used to recalculate the gaze point presented on the control screen.

### 3.4. Automatic and Manual Feedback

Sometimes, the eye tracker data may not be calibrated sufficiently and it may be difficult for the system to automatically produce feedback when a child looks directly at the stimulus. Therefore, the operator has the opportunity to produce the feedback manually. If the operator notices the child's reaction to the stimulus (utilizing the gaze-rose), he/she may click on it. It adds the point to the calibration model and, in the same time, triggers the action attached to this stimulus (sound or movement).

### 3.5. Post Hoc Analysis

All events during every session are recorded in a specific format by the storage module meant to keep data for further analysis. The recording includes eye tracker output, video registered by the camera, and screenshots of stimulus screen. The developed software provides the functionality facilitating the eye movement analysis by means of charts, scan-paths, and heatmaps [22]. The last two techniques use eye movement events—fixations and saccades. The first is defined as a movement when a person's eyes are almost stable and in the process of acquiring information from a scene. The other one is a quick movement between two fixations [15, 23]. In the case of the presented system, the IDT algorithm has been used for these event detections [24]. The algorithm is based on measuring dispersion between subsequent eye positions. If the dispersion is below a specified threshold, the gaze is considered to belong to a fixation. The presented software uses 0.5-degree threshold.

## 4. Results and Discussion

**Figure 6:**
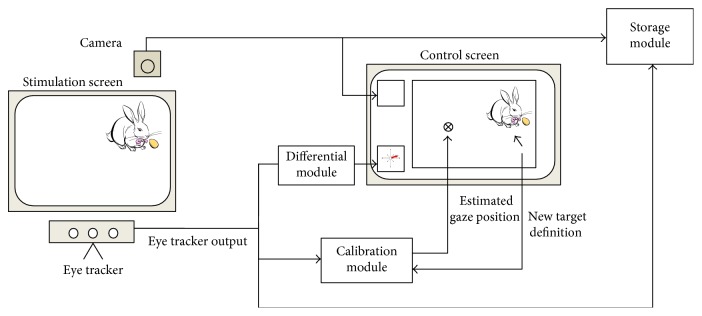
The general schema of the VisDat system.

The described system, the general schema of which is provided in Figure 6, was tested with the usage of various stimuli. The stimuli differed in the form, filling, and type of feedback they produced in a response to children's reaction, which included the following:

- Changing shape
- Changing color
- Emitting sounds
- Animated

### 4.1. Stimulus Examples

**Figure 7:**
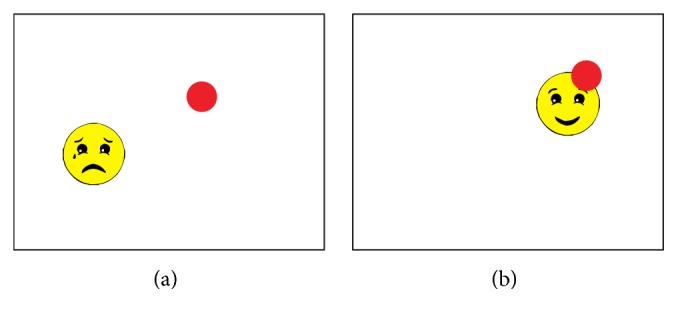
Face stimulus—when the subject is not looking at it (a) and when the subject gazes at the face (b). The red dot visible on both screen shots shows the gaze point.

An example of the first stimulus is a simplified image of a face moving on the screen (Figure 7). The face expression is sad until a child looks at the face, which makes the face smile.

**Figure 8:**
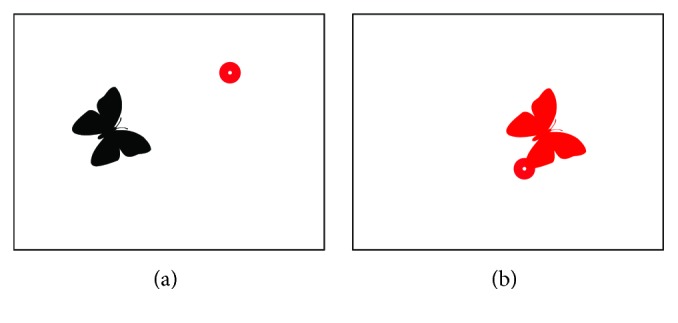
Butterfly stimulus. Image when subject is not looking at the butterfly is presented in the left screenshot (a). Image when subject is looking at the stimulus is presented in the right screenshot (b). The red dot visible on both screenshots shows the gaze point.

Some children are very sensitive to color changes. An example of a stimulus for them is presented in Figure 8. The butterfly changes its color from black to red when a subject looks at it.

**Figure 9:**
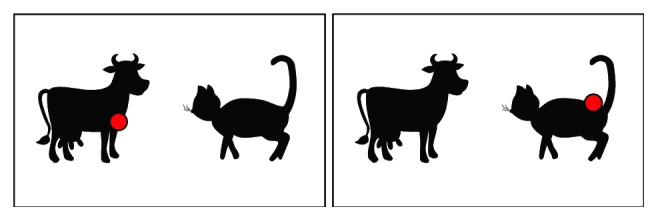
Animals stimulus. When subject is looking at the left animal, a proper sound is emitted (cow's moo in this example). When subject looks at the right animal, another sound is emitted (cat's meow).

An ability to emit sounds gives the system the opportunity to stimulate children not only visually. According to therapists, it is very important to teach children that a combination of different types of stimuli may occur. An example of such stimulus is presented in Figure 9. When a child looks at one of the animals, the appropriate sound is emitted.

**Figure 10:**
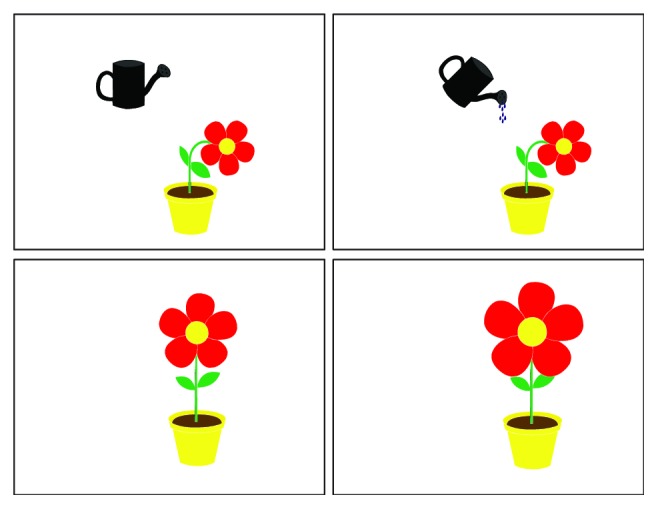
A flower in a pot and a watering can. At the beginning, the flower is withered. When a child looks at the watering can, the animation presenting watering the flower is invoked, which results in the flower straightening. If a gaze is focused on the flower, its petals grow.

By means of the stimulus editor, it is also possible to prepare more sophisticated stimuli including simple animations. An example of such an animated feedback is presented in Figure 10. The task of this stimulus is to introduce some objects' movements caused by a determined gaze. If it is placed on the watering can, it changes its position for watering flower. Directing eyes towards the flower changes its shape.

### 4.2. Visualization Examples

**Figure 11:**
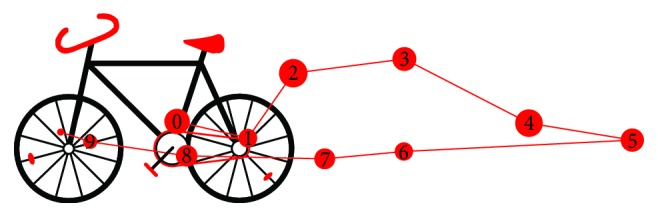
A bike stimulus with the registered eye movement signal. Red numbered dots represent fixations— movement when the eyes of the subject were still, looking at one particular point.

The experiment presented in this section involved an animated stimulus. During the experiment, a bike moved on the screen from right to left and back. The child was supposed to follow the moving object with eyes. The eye tracking data was recorded during the experiment and could be used for post hoc analysis.  Figure 11 presents the stimulus together with a part of the scan-path recorded for one child. It is visible that the child's eyes followed the object.

**Figure 12:**
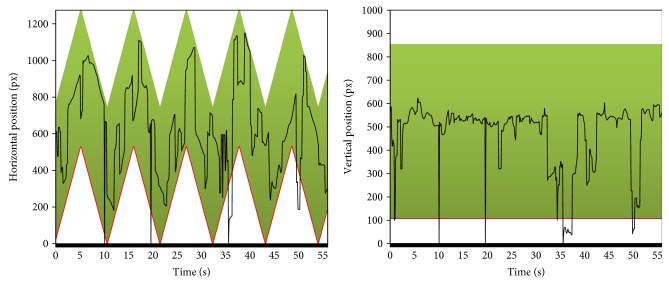
The eye position charts in both directions. The green belt represents the current position of the stimulus.

A more informative visualization is presented in Figure 12. It presents eyes' positions separately in horizontal and vertical directions compared to the current location of the bike stimulus (the colored belt). It is evident that the child's eyes followed the object moving horizontally.

**Figure 13:**
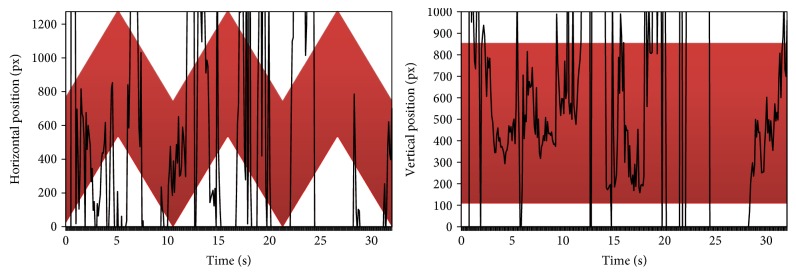
The eye position charts in both directions. The red belt represents the current position of the stimulus.

Figure 13 presents the same visualization, but for another child with more severe disparities. This time the child was able to follow the object only at the very beginning of the presentation, and even then, the eye movement signal is very instable and disappears for some periods.

More examples of the system usage are discussed in [19].

The explicit assessment of the proposed system is infeasible in a short perspective, because its effectiveness strongly depends on child's age, impairment, and its general health conditions influencing the regularity of therapy experiments. The first and basic possibility is a careful observation of a child by its therapist, who knows the child best. Additionally, we can point out some factors, which can help to confirm our observation, namely, time to the first gaze on a stimulus, increased duration of fixations on a stimulus, longer pursuit of a stimulus, or more precise saccadic movements. The first of the aforementioned metrics may indicate better perception of the objects. The second and third ones may provide evidence of the improved ability to keep attention and of better functioning of oculomotor muscles. Finally, reaching a stimulus position more precisely may reveal both better objects' recognition and progress of the oculomotor system work.

All these metrics collected during therapy sessions may be collated and compared on various charts providing convenient way for reasoning about therapy results and vision advances.

## 5. Discussion and Conclusions

The children with communication imparities require special care and treatment, because it is difficult to solve many everyday issues due to the problems with articulating them. Thus, life of such children is hard not only because of health issues but also due to the fact that their needs are not properly met.

The solution presented in this paper is a step forward towards alleviating communication difficulties and was meant as a tool providing the objective assessment of child's vision quality and areas of interest. This was achieved by the application of the affordable eye tracker, by which the developed system might become available for a wider group of people. However, this device does not suffice in case of children with imparities as they do not cooperate. For this reason, other solutions had to be elaborated, including the application working on two displays, implicit calibration procedure, and multimedia simulations responding automatically to the expected eye movement.

Preliminary experiments conducted by means of the proposed system corroborated the discussed solution's ability to become supportive in revealing children's visual performance. However, it must be emphasized that this is not the only function of this tool. Ascertaining children's sight introduces possibility of undertaking an appropriate therapy, which may be realized within the presented environment. The usage of the same stimulus or its continuous adjustment to a particular child constantly stimulates the brain and oculomotor system. This functionality has been achieved due to the convenient mechanism of the stimulus set extension, easy to perform for each system operator. This feature offers other opportunities for the system utilization not only for children but also for any “difficult” subjects who cannot or do not want to cooperate, such as adult people with various diseases including, for instance, Parkinson's disease, alcoholic disease, or even anorexia.

Currently, the authors intend to carry out detailed clinical trials of the created system in cooperation with the Association for Children with Developmental Dysfunctions BRUNO located in Rzeszow, Poland.
